# Interactions of antiretroviral drugs with the SLC22A1 (OCT1) drug transporter

**DOI:** 10.3389/fphar.2015.00078

**Published:** 2015-04-10

**Authors:** Darren M. Moss, Neill J. Liptrott, Marco Siccardi, Andrew Owen

**Affiliations:** Department of Molecular and Clinical Pharmacology, University of LiverpoolLiverpool, UK

**Keywords:** HIV, SLC22A1, OCT1, antiretrovirals, efavirenz, darunavir

## Abstract

The SLC22A1 influx transporter is expressed on the basolateral membrane of hepatocytes and is involved in the excretion of numerous cations. Inhibition of SLC22A1 by several antiretrovirals, such as the protease inhibitor darunavir, has not previously been determined. In order to better understand and predict drug-SLC22A1 interactions, a range of antiretrovirals were screened for SLC22A1-associated inhibition and transport. Stable SLC22A1-expressing KCL22 cells were produced previously by nucleofection. Control KCL22 cells were transfected with the empty vector pcDNA3.1. Accumulation of tetraethylammonium (5.5 μM, 30 min) was determined in SLC22A1-expressing and mock-transfected cells with and without 50 μM of SLC22A1 inhibitor prazosin, or 50 μM of each antiretroviral drug. SLC22A1 IC_50_ values for efavirenz, darunavir, and prazosin were determined. Cellular accumulation of efavirenz and darunavir was also assessed in SLC22A1-expressing KCL22 cells and reversibility of this accumulation was assessed using prazosin. Tetraethylammonium accumulation was higher in SLC22A1-expressing cells compared to mock-transfected cells (10.6 ± 0.8 μM vs. 0.3 ± 0.004 μM, *p* = 0.009) and was significantly reduced in SLC22A1-expressing cells when co-incubated with all antiretrovirals tested except atazanavir, lamivudine, tenofovir, zidovudine, and raltegravir. Particularly noticeable was the predominance of SLC22A1 inhibitors in the protease inhibitor and non-nucleoside reverse transcriptase inhibitor classes. Absolute SLC22A1 IC_50_ values for efavirenz, darunavir, and prazosin were 21.8, 46.2, and 2.8 μM, respectively. Efavirenz accumulation was higher in SLC22A1-expressing cells compared to mock-transfected cells (17% higher, *p* = 0.009) which was reversed using prazosin, whereas no difference was observed for darunavir (*p* = 0.86). These data inform the mechanistic basis for disposition, drug-drug interactions and pharmacogenetic candidate gene selection for antiretroviral drugs.

## Introduction

Eukaryotic drug transporting proteins play an important role in the absorption, distribution, and elimination of numerous antiretrovirals used in Human Immunodeficiency Virus (HIV) therapy. For example, the efflux transporter ABCB1 is expressed at several important cellular barriers in the body and is capable of transporting the protease inhibitors saquinavir (Janneh et al., 2005), ritonavir (Kim et al., 1998), indinavir (Lee et al., 1998), nelfinavir (Choo et al., 2000), amprenavir (Choo et al., 2000), lopinavir (Janneh et al., 2007), atazanavir (Bousquet et al., 2008), tipranavir (Orman and Perry, 2008), and darunavir (Kwan et al., 2009) *in vitro*. Antiretroviral drugs are also known to interact with influx transporters, including members of the organic anion and cation transporters of the SLCO and SLC22A gene subfamilies (Roth et al., 2012). The organic cation transporter SLC22A1 (also called Organic Cation Transporter 1, or OCT1) is predominantly expressed in the liver and is localized to the basolateral membrane of hepatocytes (Nies et al., 2008) where it mediates the uptake of substrates from the blood and facilitates drug elimination. Expression of SLC22A1 is also detected in other tissues, including the immunological cells where HIV can replicate, such as lymphoid mononuclear cells (Bleasby et al., 2006; Jung et al., 2008). The functionality of SLC22A1 has been shown to have clinically relevant consequences. As an example, the antidiabetic drug metformin is transported into the liver by SLC22A1, where it elicits a pharmacological effect. If SLC22A1 activity is reduced, this can result in inhibiting drug penetration into the liver and therefore reducing the effectiveness of treatment (Zhou et al., 2009). The SLC22A transporters play a role in regulating exposure to several antiretroviral drugs, particularly the nucleoside reverse transcriptase inhibitors (NRTIs) (Takeda et al., 2002), and SLC22A1 activity is inhibited *in vitro* by nelfinavir and ritonavir (Jung et al., 2008). However, for certain newer drugs (e.g., darunavir, lopinavir, and etravirine), the ability to inhibit and be transported by SLC22A1 has yet to be determined. The rationale of this study was to determine the inhibitory potential across antiretroviral classes for SLC22A1-mediated tetraethylammonium transport. This was used to confirm past data and to investigate the effects of previously un-assessed antiretrovirals such as darunavir, lopinavir, and etravirine. The SLC22A1-mediated transport of darunavir and efavirenz, which both showed moderate inhibition of SLC22A1 activity in initial screens, has not previously been investigated and therefore this was also assessed. This information will aid in our understanding of factors responsible for antiretroviral disposition, and may provide explanations and possible solutions for drug interactions involving antiretrovirals and co-administered substrates of SLC22A1.

## Methods

### Chemicals and materials

[^14^C]Tetraethylammonium (specific activity = 55 mCi/mmol) and [^3^H]efavirenz (specific activity = 5 Ci/mmol) were purchased from American Radiolabelled Chemicals (Missouri, USA). Lopinavir was a gift from Abbott (Illinois, USA). Raltegravir sodium salt was a gift from Merck (New Jersey, USA). Etravirine was a gift from Janssen (Buckinghamshire, UK). [^14^C]Darunavir (specific activity = 39.19 mCi/mmol), non-radiolabelled darunavir and non-radiolabelled rilpivirine were gifts from Tibotec (Mechelen Belgium). Atazanavir was a gift from Bristol-Myers Squibb (New York, USA). Nevirapine was a gift from Boehringer Ingelheim (Berkshire, UK). Ritonavir was a gift from Abbott (Illinois, USA). Tenofovir, efavirenz and lamivudine were purchased from Toronto Research Chemicals (Toronto, Canada). Amprenavir was a gift from GlaxoSmithKline (Middlesex, UK). Ultima Gold scintillation fluid was purchased from Perkin Elmer (Boston, USA). All other drugs and reagents were obtained from Sigma (Poole, UK).

### Culture of mock-transfected and SLC22A1-overexpressing KCL22 cells

SLC22A1-overexpressing chronic myeloid leukemia (KCL22) cells and mock-transfected KCL22 cells were produced by Athina Giannoudis, Royal Liverpool University Hospital, Liverpool, UK, as previously described (Giannoudis et al., 2008). SLC22A1-overexpressing KCL22 cells were created by transfecting pcDNA-hSLC22A1 plasmid into KCL22 cells by nucleofection. Similarly, mock-transfected KCL22 cells were created by transfecting the empty vector pcDNA3.1 into cells by nucleofection. To create stable cell lines, transfected cells were subjected to neomycin and surviving clones were selected. Non-transfected KCL22 cells are known to express only a low level of SLC22A1 in comparison to other chronic myelogenous leukemia cell lines and were therefore suitable for use in the current study (Thomas et al., 2004). KCL22 cells were maintained in cell culture medium [Roswell Park Memorial Institute medium (RPMI), 10% [vol/vol] fetal calf serum (FCS)] prior to experiments in a CO_2_ incubator (37°C, 5% CO_2_). All cell culture procedures were performed in a sterile environment.

### Determination of tetraethylammonium accumulation in SLC22A1-expressing KCL22 cells co-incubated with antiretroviral drugs

SLC22A1-overexpressing KCL22 cells and mock-transfected KCL22 cells of a constant cell density (1 mL, 2.5 × 10^6^ cells/mL) were incubated (37°C, 5% CO_2_) for 30 min in cell culture medium (RPMI, 10% [vol/vol] FCS) containing SLC22A1 substrate [^14^C]tetraethylammonium (5.5 μM, 0.3 μCi/mL). Separate incubations were undertaken where SLC22A1-overexpressing KCL22 cells were preincubated for 30 min prior to the substrate addition with cell culture medium (RPMI, 10% [vol/vol] FCS) containing one of a selection of co-incubated drugs, which included either 50 μM of SLC22A1 inhibitors prazosin, 50 μM of the immunosuppressant cepharanthine, or 50 μM of each antiretroviral drug, which were also included during the 30 min of substrate incubation. It should be noted that prazosin is also capable of inhibiting SLC22A3 (Hayer-Zillgen et al., 2002), although this is unlikely to play into the experiment as SLC22A3 is not excepted to be significantly expressed in KCL22 cells, which are derived from chronic myeloid leukemia cells (Bleasby et al., 2006). The antiretroviral drugs examined as potential SLC22A1 inhibitors were atazanavir, lopinavir, amprenavir, indinavir, darunavir, ritonavir, nelfinavir, lamivudine, tenofovir, zalcitabine, abacavir, zidovudine, stavudine, etravirine, nevirapine, rilpivirine, efavirenz, and raltegravir. To assess toxicity of inhibitor in each sample, following the incubation 5 μL was removed and added to 5 μL trypan blue 0.4% for cell viability assessment using a Countess™ (Thermo Fisher, MA) automated cell counter. Samples were discarded if cell viability was less than 80% of the viability determined in inhibitor-free control samples. The remaining incubation was centrifuged (800 × g, 1°C, 1 min), supernatant fraction was discarded and the cells were washed with ice-cold HBSS and centrifuged (800 × g, 1°C, 1 min). This HBSS wash was repeated a total of three times, after which the HBSS was discarded and 100 μL tap water was added to lyse the cells. The incubations were vortexed for 5 min and samples were added to scintillation vials. Four milliliters of scintillation fluid was added to scintillation vials, which were then loaded into a liquid scintillation analyzer (TRI-CARB®). Using intracellular radioactivity readings, intracellular tetraethylammonium concentrations were determined (μM ± SD, assuming 1 pL volume per cell).

### Determination of the SLC22A1 IC_50_ of efavirenz and darunavir

Prior to this study, there has been no published data on the inhibition of SLC22A1 by the protease inhibitor darunavir. Furthermore, efavirenz did not show SLC22A1 inhibition in a previous publication, which contradicted our data (Jung et al., 2008). Therefore, the inhibitory potential (IC_50_) of darunavir and efavirenz for SLC22A1-mediated transport was assessed in more detail to confirm results. Accumulation of tetraethylammonium (5.5 μM, 0.3 μCi/mL) was determined when cells were co-incubated for 30 min with a log range concentration of either efavirenz or darunavir (0, 1, 2.5, 5, 10, 25, 50, 100 μM). A parallel experiment was performed using prazosin as a positive control inhibitor of SLC22A1-mediated tetraethylammonium transport. To assess toxicity of inhibitor in each sample, cellular integrity was assessed using the trypan blue exclusion test as described previously. Following incubation, intracellular concentrations were analyzed as described in the previous section and data was plotted using Prism 5. Non-linear regression analysis was used to calculate relative IC_50_ (the amount of drug needed to achieve 50% SLC22A1 inhibition as determined from the maximum and minimum extremes of the non-linear regression plot) and absolute IC_50_ (the amount of drug needed to achieve 50% SLC22A1 as determined from the maximum of the non-linear regression plot and 0% accumulation).

### Determination of accumulation of efavirenz and darunavir in SLC22A1-overexpressing KCL22 cells

Mock transfected KCL22 cells and SLC22A1-overexpressing KCL22 cells of a constant cell density were incubated (1 mL, 2.5 × 10^6^ cells/mL 37°C, 5% CO_2_) in cell culture medium (RPMI, 10% [vol/vol] FCS, 30 min) containing either efavirenz (20 μM), darunavir (20 μM) or SLC22A1 control substrate tetraethylammonium (1 μM). A parallel incubation was undertaken where SLC22A1-expressing KCL22 cells were preincubated prior to the substrate addition in cell culture medium containing the potent SLC22A1 inhibitor, prazosin (RPMI, 10% [vol/vol] FCS, 100 μM prazosin, 30 min) and prazosin was also included during the 30 min substrate incubation. To assess toxicity of inhibitor in each sample, cellular integrity was assessed using the trypan blue exclusion test as described previously. Following incubation, intracellular concentrations were analyzed as described above. Using intracellular radioactivity readings, the amount of drug in mock transfected cells was compared to SLC22A1-overexpressing KCL22 cells (% ± SD). The amount of drug in SLC22A1-overexpressing KCL22 cells incubated with prazosin was also compared to SLC22A1-overexpressing KCL22 cells (% ± SD).

### Statistical analyses

Data were analyzed using SPSS 20 for Windows. IC_50_ curves were generated using Prism 5 for Windows. The Mann Whitney *U*-test was used to evaluate significance for all data. A two-tailed *p*-value of <0.05 was accepted as being statistically significant.

## Results

### Accumulation of tetraethylammonium in SLC22A1-overexpressing KCL22 cells co-incubated with antiretroviral drugs

**Figure 1:**
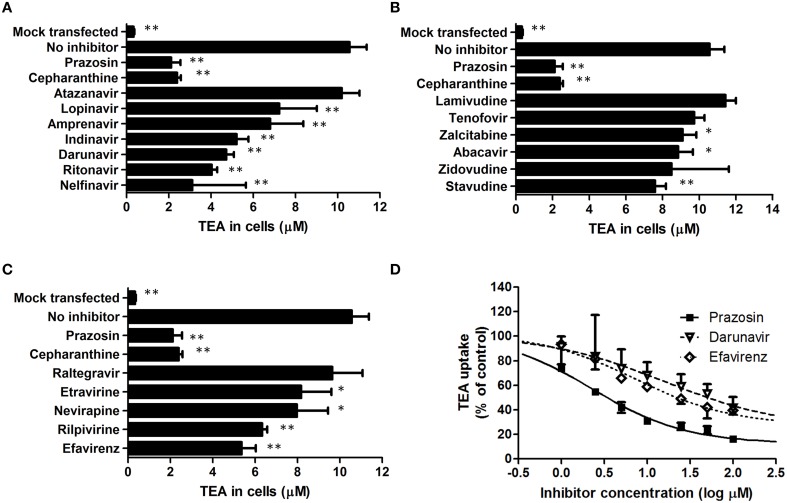
**The effects of protease inhibitors (A), nucleoside reverse transcriptase inhibitors (B), non-nucleoside reverse transcriptase inhibitors (C) and the integrase inhibitor raltegravir (C) on the accumulation of tetraethylammonium in KCL22 cells**. Results are given as tetraethylammonium concentrations in cells after 30 min incubation (μM, *n* = 9 replicates in SLC22A1-overexpressing KCL22 cells incubated with tetraethylammonium, *n* = 3 experimental replicates in mock transfected cells incubated with tetraethylammonium and *n* = 3 experimental replicates in SLC22A1-overexpressing KCL22 cells incubated with both tetraethyammonium and test compound, * *p* < 0.05, ** *p* < 0.01) ± SD. The IC_50_ values of prazosin, efavirenz, and darunavir were determined in further experiments **(D)**. Results are given as tetraethylammonium concentrations in cells after 30 min incubation (μM, *n* = 3 experimental replicates) ± SD.

The inhibition of SLC22A1-mediated tetraethylammonium transport by HIV PIs (Figure 1A), NRTIs (Figure 1B), NNRTIs (Figure 1C) and the integrase inhibitor raltegravir (Figure 1C) was determined in transfected SLC22A1-overexpressing KCL22 cells and mock transfected control KCL22 cells. Prazosin was included as a control SLC22A1 inhibitor for validation, and cepharanthine was assessed for SLC22A1 inhibition. Results are given as tetraethylammonium concentration in cells after 30 min incubation (μM, *n* = 9 replicates in SLC22A1-overexpressing KCL22 cells incubated with tetraethylammonium, *n* = 3 experimental replicates in mock transfected cells incubated with tetraethylammonium and *n* = 3 experimental replicates in SLC22A1-overexpressing KCL22 cells incubated with both tetraethyammonium and test compound) ± SD. Tetraethylammonium cellular accumulation was significantly higher in SLC22A1-overexpressing KCL22 cells compared to mock transfected control KCL22 cells (10.6 ± 0.8 μM vs. 0.3 ± 0.04 μM, *p* = 0.009). Tetraethylammonium cellular accumulation was significantly reduced in SLC22A1-expressing KCL22 cells when cells were co-incubated with 50 μM test compound lopinavir (7.2 ± 1.8 μM, *p* = 0.009), amprenavir (6.8 ± 1.6 μM, *p* = 0.009), indinavir (5.2 ± 0.6 μM, *p* = 0.009), darunavir (4.7 ± 0.4 μM, *p* = 0.009), ritonavir (4.0 ± 0.3 μM, *p* = 0.009), nelfinavir (3.1 ± 2.5 μM, *p* = 0.009), zalcitabine (9.1 ± 0.7 μM, *p* = 0.048), abacavir (8.8 ± 0.8 μM, *p* = 0.036), stavudine (7.6 ± 0.6 μM, *p* = 0.009), etravirine (8.2 ± 1.4 μM, *p* = 0.036), nevirapine (8.0 ± 1.5 μM, *p* = 0.018), rilpivirine (6.3 ± 0.2 μM, *p* = 0.009), efavirenz (5.4 ± 0.7 μM, *p* < 0.009), cepharanthine (2.4 ± 0.2 μM, *p* = 0.009), or control SLC22A1 inhibitor prazosin (2.1 ± 0.4 μM, *p* = 0.009). Tetraethylammonium cellular accumulation was not significantly altered in SLC22A1-expressing KCL22 cells when cells were co-incubated with 50 μM atazanavir (10.2 ± 0.8 μM, *p* = 0.839), lamivudine (11.4 ± 0.6 μM, *p* = 0.145), tenofovir (9.7 ± 0.6 μM, *p* = 0.145), zidovudine (8.5 ± 3.1 μM, *p* = 0.1), or raltegravir (9.6 ± 1.4 μM, *p* = 0.373), indicating that these antiretrovirals are unlikely to inhibit SLC22A1 activity at therapeutic concentrations.

### Inhibitory potential of efavirenz and darunavir in SLC22A1-overexpressing KCL22 cells

The IC_50_ of SLC22A1-mediated tetraethylammonium transport using a concentration range of efavirenz, darunavir, or prazosin was determined (Figure 1D). The concentration-inhibition relationship curves of efavirenz and darunavir appeared to plateau before complete inhibition of SLC22A1 was achieved, which may be due to drug solubility limitations or non-competitive SLC22A1 inhibition. Therefore, plots were used to calculate both relative and absolute IC_50_ values (Table 1). Control SLC22A1 inhibitor prazosin achieved an 84% reduction in cellular tetraethylammonium accumulation at 100 μM and a relative IC_50_ value of 2.3 μM, which is similar to values found in published literature (Minematsu et al., 2010).

**Table 1:** **Maximum observed SLC22A1 inhibition and calculated relative and absolute IC_50_ values for darunavir, efavirenz, and control SLC22A1 inhibitor prazosin**.

**Inhibitor**	**TEA accumulation when 100 μM inhibitor used (%compared to inhibitor-free control)**	**Relative IC_50_ (μM)**	**Absolute IC_50_ (μM)**
Prazosin	16.1	2.3	2.8
Darunavir	41.3	15.9	46.2
Efavirenz	39.7	7.4	21.8

The relative IC_50_ is the amount of drug needed to achieve 50% SLC22A1 inhibition as determined from the maximum and minimum extremes of the non-linear regression plot. The absolute IC_50_ is the amount of drug needed to achieve 50% SLC22A1 as determined from the maximum of the non-linear regression plot and 0% accumulation.

### Accumulation of efavirenz and darunavir in SLC22A1-overexpressing KCL22 cells co-incubated with prazosin

**Figure 2:**
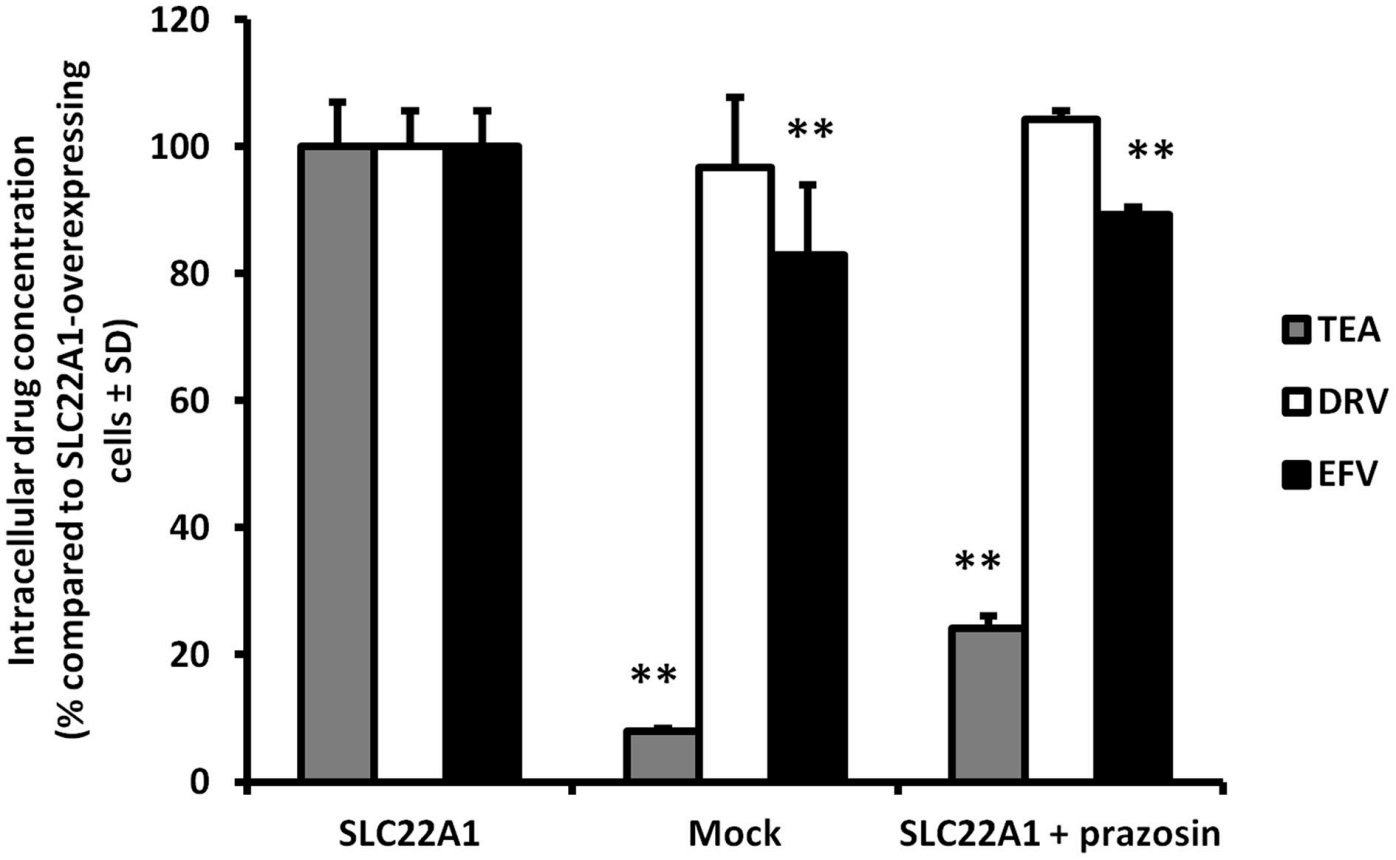
**The accumulation of tetraethylammonium, efavirenz, and darunavir in mock-transfected KCL22 cells, SLC22A1-overexpressing KCL22 cells and SLC22A1-overexpressing cells co-incubated with prazosin**. The amount of drug in mock transfected cells and SLC22A1-overexpressing KCL22 cells subjected to prazosin were compared to the amount of drug in SLC22A1-overexpressing KCL22 cells (% compared to SLC22A1-overexpressing cells ± SD, *n* = 9 replicates in SLC22A1-overexpressing KCL22 cells incubated with test substrate, *n* = 3 experimental replicates in mock transfected cells incubated with test substrate and *n* = 3 experimental replicates in SLC22A1-overexpressing KCL22 cells incubated with both test substrate and prazosin). ** *p* < 0.01.

Accumulation of efavirenz and darunavir in SLC22A1-overexpressing and mock-transfected KCL22 cells, and the influence of SLC22A1 inhibitor prazosin, were determined (Figure 2) (% accumulation of test substrate compared to accumulation in SLC22A1-overexpressing cells, *n* = 9 replicates in SLC22A1-overexpressing KCL22 cells incubated with test substrate, *n* = 3 experimental replicates in mock transfected cells incubated with test substrate and *n* = 3 experimental replicates in SLC22A1-overexpressing KCL22 cells incubated with both test substrate and prazosin) ± SD. Tetraethylammonium was used to validate the experiment and showed significantly decreased amounts in mock-transfected cells (92% less, *p* = 0.009) and SLC22A1-overexpressing cells subjected to prazosin (76% less, *p* = 0.009), when compared to SLC22A1-overexpressing cells. Efavirenz showed a minor but significantly lower amount in mock-transfected cells (17% less, *p* = 0.009) and SLC22A1-overexpressing cells subjected to prazosin (11% less, *p* = 0.009), when compared to SLC22A1-overexpressing cells. Darunavir did not accumulate to a different extent in mock-transfected cells (*p* = 0.86) or SLC22A1-overexpressing cells subjected to prazosin (*p* = 0.21), when compared to SLC22A1-overexpressing cells.

## Discussion

Overall, SLC22A1 inhibition was achieved using numerous antiretrovirals: all protease inhibitors apart from atazanavir were able to reduce tetraethylammonium accumulation. These findings agree with previous data, where saquinavir, indinavir, ritonavir, and nelfinavir were able to reduce tetraethylammonium accumulation in SLC22A1-overexpressing Hela cells (Zhang et al., 2000) and atazanavir was found to have no SLC22A1-inhibiting capability *in vitro* (Jung et al., 2008). As inhibition of SLC22A1 by darunavir has not been previously investigated and the inhibition observed was more substantial than most protease inhibitors, a concentration-inhibition relationship was established in this study. Although darunavir showed inhibition of SLC22A1, it should be noted that the maximum darunavir plasma concentrations in patients taking 600 mg/100 mg darunavir/ritonavir (8.8 μM) is lower than the relative (15.9 μM) and absolute (46.2 μM) SLC22A1 IC_50_ value determined in this study (Sekar et al., 2008). Generally, the NRTIs did not inhibit SLC22A1 or only showed minor inhibition. Lamivudine did not inhibit SLC22A1 and this contradicts previous published data which showed significant inhibition (Jung et al., 2008). However, Jung et al used 1-methyl-4-phenylpyridinium as the control SLC22A1 substrate and this could explain the conflicting data. Indeed, it may suggest the existence of separate substrate binding sites on SLC22A1, only one of which is able to be inhibited by lamivudine. Also, the previous study utilized kidney-derived HEK-293 cells, which may have naturally expressed other transporters of 1-methyl-4-phenylpyridinium, which were able to be inhibited by lamivudine. The non-nucleoside reverse transcriptase inhibitors all showed some level of SLC22A1 inhibition, although inhibition by etravirine and nevirapine was minor (22.5% and 24.5% reduction in cellular accumulation, respectively, when using 50 μM drug). Efavirenz and rilpivirine showed a more substantial level of SLC22A1 inhibition (50.0% and 41.2% reduction in cellular accumulation, respectively, when using 50 μM drug). Efavirenz did not show SLC22A1 inhibition in a previous publication, which contradicts our data (Jung et al., 2008). However, Jung et al used 1-methyl-4-phenylpyridinium as the control SLC22A1 substrate, and efavirenz was assessed only at a concentration of 5 μM. Also, incubations in the study were only 1 min (compared to 30 min in the current study) and time-dependent inhibition may be apparent. To investigate further, SLC22A1 IC_50_ values were determined for efavirenz. It should be noted that the maximum plasma concentration of efavirenz (12.7 μM) is lower than the absolute SLC22A1 IC_50_ value (21.8 μM), but higher than the relative SLC22A1 IC_50_ value (7.4 μM), determined in this study (Villani et al., 1999). The immunosuppressant cepharanthine was also found to be an inhibitor of SLC22A1, which is a novel discovery. The small extent of SLC22A1-mediated efavirenz transport may suggest a minor role for SLC22A1 in efavirenz disposition but it is not possible to extrapolate these *in vitro* data directly to the *in vivo* scenario. To assess this more completely, it may be possible to perform a study in animals where the effects SLC22A1-knockdown or inhibition was determined on efavirenz pharmacokinetics. SLC22A1 pharmacogenetic studies may be warranted in patients taking efavirenz in order to determine the relevance of SLC22A1 polymorphisms on efavirenz pharmacokinetics. Additionally, this information could potentially be utilized to establish more effective efavirenz treatment strategies in populations with relevant SLC22A1 polymorphisms.

### Conflict of interest statement

The authors declare that the research was conducted in the absence of any commercial or financial relationships that could be construed as a potential conflict of interest.
